# F233. NEGATIVE SYMPTOMS ARE INDEPENDENT MODERATOR FACTORS OF TREATMENT RESISTANT SCHIZOPHRENIA EFFECTS ON MULTIPLE CLINICAL, PSYCHOPATHOLOGICAL, COGNITIVE AND PSYCHOSOCIAL VARIABLES

**DOI:** 10.1093/schbul/sby017.764

**Published:** 2018-04-01

**Authors:** Felice Iasevoli, Benedetta Altavilla, Camilla Avagliano, Annarita Barone, Luigi D’Ambrosio, Marta Matrone, Danilo Notar Francesco, Eugenio Razzino, Andrea de Bartolomeis

**Affiliations:** University of Naples “Federico II”

## Abstract

**Background:**

Negative symptoms (NSs) are more severe in Treatment Resistant Schizophrenia (TRS) than Antipsychotic Responder Schizophrenia (ARS) patients. NSs are predictors of outcomes of neurological soft signs and functional capacity in TRS but not in ARS patients. The scope of this work is to clarify whether NSs effects are integral to or independent from the TRS diagnosis in our sample of patients.

**Methods:**

70 out of 206 eligible putative TRS and ARS patients were included (enrollment still ongoing). Patients were tested by the Neurological Evaluation Scale (NES); the CGI-S; the PANSS; the Heinrichs’ Quality of Life Scale (QLS); the UCSD Performance-Based Skills Assessment (UPSA); the Personal and Social Performance (PSP) scale and Specific Level of Functioning (SLOF). Patients were subdivided in NSHigh (severe NSs) and NSLow (mild NSs) based on ROC curve-derived cut-off.

**Results:**

At the Student’s t test, NSHigh had significantly lower scores than NSLow patients on: Verbal Fluency; QLS score; PSP score; UPSA Financial, Communication, and Family Skills; UPSA total score; all SLOF areas (except Area4). NSHigh patients had significantly higher scores than NSLow patients on CGI-S; PANSS Positive and General Psychopathology Subscale scores; and NES score.

Distribution of NS patients was significantly different between TRS/ARS diagnostic groups, as NSHigh patients were significantly more frequent in the TRS group (Pearson chi square: □1=5.51, p=.001). Notably, mean PANSS Negative Subscale scores were significantly higher in TRS compared to ARS patients (Student’s t: F1,58=2.84, p=.006).

Since multiple variables found to be significantly different in NSHigh vs. NSLow patients were also significantly different between TRS and ARS patients, the question arises whether the significant differences found between diagnostic groups may depend on the higher percentage of patients with more severe NSs in the TRS group. Therefore, a two-way ANOVA was carried out with dichotomous NS and Diagnosis variables as the independent variables. Outcomes on multiple clinical variables were significantly different among groups. A NS*Diagnosis interaction effect was found for NES score (F1,58=4.32, p=.042, Visuospatial Memory, UPSA Transportation skills, and SLOF Area1. In all these cases, NSHigh/TRS patients performed significantly worse than the other patient groups; in the case of NES score, NSHigh/TRS patients score significantly higher than the other groups. Independent effect of either NSs or Diagnosis were also found for multiple variables, suggesting that NSs and Diagnosis may interact but their effects are not completely overlapping. To have a more deepen comprehension of NS effects on diagnosis, we carried out a moderator regression analysis and an ANCOVA analysis that further confirmed the finding that NSs’ mediate Diagnosis effects on a number of clinical outcomes.

Given that NSs largely affect clinical variables, we asked which distinct symptom may exert the greater impact on each of these variables. Therefore, we carried out a including the seven PANSS Negative Subscale items as the independent variables. The items that explained the highest variance in clinical variables were mostly Stereotyped Thinking (N7), Passive Social Withdrawal (N4), and Difficulty in Abstract Thinking (N5).

**Discussion:**

These data suggest that NSs are both independent determinants and moderators of TRS/ARS diagnosis effect on multiple psychopathology, cognitive, and psychosocial factors. More impaired functions attributed to non-response to antipsychotics may depend on more severe NSs. However, only a subset of NSs appears to exert this action, possibly related to the multidimensional construct of these symptoms.

